# Personalized chemotherapy selection for patients with triple-negative breast cancer using deep learning

**DOI:** 10.3389/fmed.2024.1418800

**Published:** 2024-06-20

**Authors:** Xinyi Yang, Reshetov Iogr Vladmirovich, Poltavskaya Maria Georgievna, Agakina Yulia Sergeevna, Mingze He, Zitong Zeng, Yinpeng Qiang, Yu Cao, Kulikov Timur Sergeevich

**Affiliations:** ^1^I.M. Sechenov First Moscow State Medical University (Sechenov University), Moscow, Russia; ^2^Institute for Urology and Reproductive Health, Sechenov University, Moscow, Russia; ^3^Department of Faculty Surgery No. 2, Sechenov University, Moscow, Russia

**Keywords:** triple-negative breast cancer, chemotherapy, deep learning, causal inference, adjuvant therapy

## Abstract

**Background:**

Potential uncertainties and overtreatment exist in adjuvant chemotherapy for triple-negative breast cancer (TNBC) patients.

**Objectives:**

This study aims to explore the performance of deep learning (DL) models in personalized chemotherapy selection and quantify the impact of baseline characteristics on treatment efficacy.

**Methods:**

Patients who received treatment recommended by models were compared to those who did not. Overall survival for treatment according to model recommendations was the primary outcome. To mitigate bias, inverse probability treatment weighting (IPTW) was employed. A mixed-effect multivariate linear regression was employed to visualize the influence of certain baseline features of patients on chemotherapy selection.

**Results:**

A total of 10,070 female TNBC patients met the inclusion criteria. Treatment according to Self-Normalizing Balanced (SNB) individual treatment effect for survival data model recommendations was associated with a survival benefit (IPTW-adjusted hazard ratio: 0.53, 95% CI, 0.32–8.60; IPTW-adjusted risk difference: 12.90, 95% CI, 6.99–19.01; IPTW-adjusted the difference in restricted mean survival time: 5.54, 95% CI, 1.36–8.61), which surpassed other models and the National Comprehensive Cancer Network guidelines. No survival benefit for chemotherapy was seen for patients not recommended to receive this treatment. SNB predicted older patients with larger tumors and more positive lymph nodes are the optimal candidates for chemotherapy.

**Conclusion:**

These findings suggest that the SNB model may identify patients with TNBC who could benefit from chemotherapy. This novel analytical approach may provide debiased individual survival information and treatment recommendations. Further research is required to validate these models in clinical settings with more features and outcome measurements.

## Introduction

Breast cancer is the most prevalent malignant tumor in women worldwide (1) and the leading cause of cancer-related deaths (2). Triple-negative breast cancer (TNBC) is the most aggressive subtype of breast cancer (3), which is characterized by the absence of estrogen receptors (ERs) and progesterone receptors (PRs), as well as the lack of overexpression of human epidermal growth factor receptor 2 (HER2) (4). Patients with TNBC account for 10–20% of breast cancer cases diagnosed each year (5), and they have a higher rate of recurrence and mortality (6).

Currently, adjuvant chemotherapy is the standard of care for operable TNBC, but it is only partially effective (7). For example, the National Comprehensive Cancer Network (NCCN) guidelines only recommend adjuvant chemotherapy for patients with tumor size larger than 1 cm (beyond T1b) or pN+ (8). However, a number of studies have found that patients with T1b TNBC still benefit after receiving adjuvant chemotherapy (9, 10). In addition to tumor size, age, race, surgery, and radiation therapy are also important indicators for chemotherapy decisions (11). This indicates that the therapeutic heterogeneity of adjuvant chemotherapy cannot be ignored in the TNBC population.

The individuality of the patient should be at the core of every treatment decision (12). Estimating the average treatment effect with randomized control trials (RCTs) or observational studies that incorporate extensive statistical theories only provides a coarse summary of the distribution of a treatment effect, which may be inapplicable or even misleading at the individual level (13). Traditionally, to assess the heterogeneity of treatment and select the optimal treatment for a particular patient, researchers should continually subdivide subgroups through clinical experience to approximate an individual patient or a particular class of patients and repeatedly conduct RCTs within these subgroups. However, the traditional approach is not only very expensive and time-consuming but also ethically restrictive (14). Inferring unbiased individual treatment effects (ITEs) in observational studies is challenging because observational data can be affected by numerous biases (13). Leveraging machine learning, the ITEs can be predicted through counterfactual reasoning (13). Previous studies (15, 16) have demonstrated that the deep learning (DL)-based treatment recommendation system can effectively predict ITEs, recognize treatment heterogeneity, and select optimum treatment for the patients.

The aim of this study is to establish a set of sophisticated DL treatment guidelines. Thus, it can provide optimal adjuvant chemotherapy recommendations for TNBC patients at the individual level and help patients achieve the longest possible survival.

## Methods

### Study design and setting

This was a population-based retrospective cohort study making individualized adjuvant chemotherapy recommendations for patients with TNBC using DL. All participants in this study were included in the Surveillance, Epidemiology, and End Results (SEER) 18 database, which tracks cancer patients in 18 regions of the United States and represents approximately 27.8% of the national population (17). This study followed the Strengthening the Reporting of Observational Studies in Epidemiology reporting guidelines (18).

**Figure 1:**
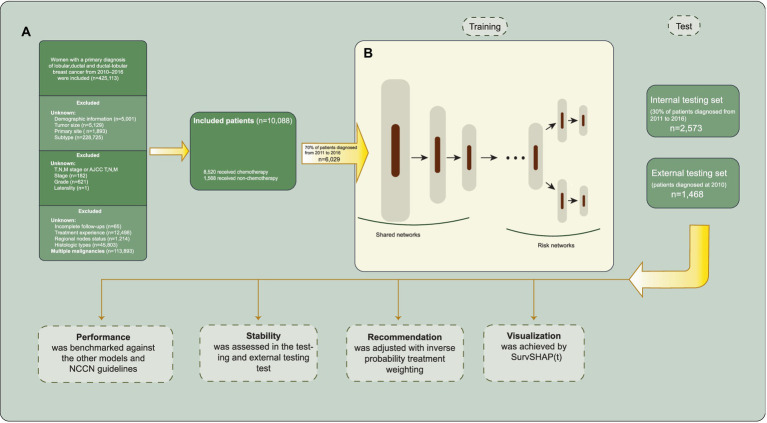
Inclusion process and model architecture. **(A)** Selection and exclusion criteria for patient inclusion. **(B)** The model architecture of Self-Normalizing Balanced (SNB) individual treatment effect for survival data.

Female patients diagnosed with ductal, lobular, or ductal-lobular carcinoma as a single primary cancer between 2010 and 2016, who underwent either breast-conserving surgery (BCS) or mastectomy, were included in the study. The exclusion criteria were as follows: (1) missing demographic information; (2) unknown HER2, ER, or PR status; (3) carcinoma *in situ*; (4) unknown laterality and bilateral breast cancer; (5) unspecified Tumor Node Metastasis (TNM) stage or tumor size; (6) unknown metastasis sites; (7) unknown axillary lymph node status; (8) uncertain whether adjuvant or neoadjuvant systemic treatment and radiotherapy was performed; (9) unknown histologic grades and types; and (10) incomplete follow-up or multiple malignancies. The inclusion process is illustrated in Figure 1A.

We collected baseline information (sex, age, race, income, and marriage status), tumor characteristics (location, size, laterality, histological grade, histologic type, and TNM stage), and treatment details (type of surgery and chemotherapy) for cases from the SEER database. Patients were excluded if any of the included clinical characteristic statuses were undocumented or missing. The primary outcomes of this study included overall survival (OS), which is the time interval from diagnosis to all-cause death. Patients who remained alive on 31 December 2020 were censored in the study. The tumor stage was determined according to the 7th American Joint Committee on Cancer Staging Manual.

### Algorithms

The T-learner adopts two models to estimate the ITEs by $ITE=\mu_{1}\left(x\right)-\mu_{0}\left(x\right)$ , where $\mu_{1}$ and $\mu_{0}$ denote the models trained on corresponding treatment populations (19). This approach is composed of two estimators trained in different treatment groups, representing different treatment hypotheses in inference. The ITE is computed by observing the difference in predictions between these two estimators, which can be any prediction model, such as the Cox proportional hazards (CPH) model. While the T-learner can exclude certain confounding factors, it remains vulnerable to inconsistent predictive performance (13) and biased treatment allocation (20) due to disparate patient numbers and imbalanced baseline characteristics in the two treatment groups. DeepSurv (21) was originally proposed to relax the linearity and normality assumptions of CPH by replacing the single-layer linear model of CPH with a multilayer perceptron (MLP). In a follow-up study (15), it was found that combining DeepSurv with the T-learner was effective in inferring ITEs.

Cox Mixtures with Heterogeneous Effects (CMHEs) (22) operate on the assumption that the cohort consists of potential subgroups with different survival scenarios. Within each risk group, the proportional hazards assumption holds a concept known as the conditional proportional hazard assumption. To maximize the representation of diverse risk groups, the expectation–maximization technique was implemented.

The Balanced Individual Treatment Effect for Survival (BITES) data (20), a semi-parametric DL survival regression model, addresses the issue of selection bias using representation-based causal inference. It contains a shared network and two risk networks (three MLPs) and uses the Integral Probability Metrics to maximize the p-Wasserstein distance of different treatment arms in the shared network, which proved to be effective in controlling imbalance for both covariate space (23) and latent representations (24). The BITES utilizes a holistic model with two output heads to replace the two separately trained estimators of the T-learner. As the BITES is trained end-to-end, it is less susceptible to inconsistencies in the number of patients between the two treatment groups.

The SNB individual treatment effect for survival data (25) integrates the T-learner and representation-based causal inference methods. The architecture of SNB is presented in Figure 1B. SNB inherits the overall architecture of BITES with MLPs replaced with self-normalizing neural networks (SNNs) (26). The neuron activations of SNNs automatically converge toward zero mean and unit variance, which in turn avoids exploding and vanishing gradients. Therefore, the feature extraction ability and robustness of SNB are significantly improved, which is expected to accurately predict the factual and counterfactual survival outcomes, thereby inferring more accurate ITEs. The shared network calculates balanced (debiased) latent representation using Smoothed Optimal Transport loss (27). Each risk network represents the corresponding treatment group, akin to a T-learner.

### Calculation of individual treatment effect

When estimating the ITEs, we can observe only one outcome per patient; the alternative scenario remains hypothetical and thus unobservable. Thus, these outcomes need to be predicted by models. The individual survival distribution is obtained with the predicted log hazard ratios and treatment-specific baseline hazards, which describe the change in survival probability over time.

We define the clinically interpretable potential outcome as the area under the survival curve for an individual over a specified period (10 years), termed restricted survival time (RST). The formula can be described as: $ITE_{RST}\left(X;t\right)=\underset{x\in X}{\sum }\left[\overset{t}{\underset{0}{\int }}{\hat{S}}_{1}\left(t|x\right)dt-\overset{t}{\underset{0}{\int }}{\hat{S}}_{0}\left(t|x\right)dt\right]$ , where t indicates the preset time horizon, x indicates the covariates, and ${\hat{S}}_{0}\left(t|x\right)$ and ${\hat{S}}_{1}\left(t|x\right)$ are the predicted survival distributions for an individual under two treatment scenarios, respectively. Individualized treatment recommendations can then be obtained based on the value of ITEs.

### Model development, validation, and treatment recommendation

We trained five models in total: SNB, BITES, Cox Mixtures with Heterogeneous Effects (CMHE) (22), DeepSurv (21), and CPH. The DeepSurv and CPH were trained and used with a T-learner structure.

Initially, we selected patients diagnosed in 2010 to serve as an external testing set concealed from the models. In the remaining data, patients were randomly allocated to a training set of 70% of the samples used for building the models; and a testing set of 30% of the samples, unseen by models, were used for evaluating the model performance. During training, we used five-fold cross-validation to tune the hyperparameters of the model; each time, the model was trained on four-fifths of the training set and validated on the remaining one-fifth of the training set. The training process will be automatically terminated if the validation loss does not decrease in 1,000 iterations. Tuned hyperparameters included the nodes and layers of MLPs or SNNs, learning rate, mini-batch size, the strength of Smoothed Optimal Transport loss (applicable for BITES and SNB), and the number of risk groups (applicable for CMHE). We did not take any missing value filling approach because there were no missing values. When feeding the models, all categorical variables that contain more than three factors were processed with one-hot encoding.

To explore the recommendation effect of models, we divided the patients into the recommended (Consis.) and anti-recommended (Inconsis.) groups based on whether the actual treatment they received was consistent with the model recommendations. The multivariate hazard ratio (HR), 10-year risk difference (RD), and the difference in the 10-year restricted mean survival time (DRMST) were calculated between Consis. and Inconsis. groups to evaluate the protective effects of models. The HR compares the relative risk of an event occurring between two groups over time; RD represents the absolute difference in event rates between two groups; and DRMST measures the change in average survival time between two groups. Overall, these metrics measure the survival advantage that following model recommendations can provide over not following them. A positive difference indicates longer survival in the treatment group. A positive RD suggests a higher event rate in the treatment group, while a negative RD indicates a lower rate. Inverse probability treatment weighting (IPTW) was used to control for baseline imbalance between the Consis. and Inconsis. groups. All models used the same ITE calculation methods. To prevent the potential that the Consis. group may have better prognostic factors, the IPTW was used to correct the baseline imbalance between the Consis. and Inconsis. groups. Demographic and tumor characteristics were adjusted, including age, race, marriage status, income, location, laterality, histology, grade, TNM stage, tumor size, and lymph node positivity. Treatment variables were not adjusted as they were measured after exposure (treatment recommendation) and may introduce unmeasured confounding (28).

To account for the effect of covariates on relative efficacy, we calculated the linear relationship between patient characteristics and ITEs (29). Considering that the SEER database contains patients originating from different regions, a mixed-effect linear regression was used to calculate this effect. It enables the model to account for and capture regional heterogeneity, thereby improving the accuracy and generalizability of the estimates (30).

### Statistical analyses

All statistical analyses were performed using R version 4.1.3 and Python version 3.8. Models were built with Python packages Pytorch 2.0.0 and scikit-survival 0.19.0, with main codes provided by the original papers cited above. We have made some improvements and integrations to the source codes, which are open source in Github: https://github.com/xinyi1999/MyPublication. In this repository, model codes, ITE calculations, and other methods are documented. Metrics were calculated using the R packages survival and rms. The IPTW was conducted using the R package ipw. The mixed-effect linear regression was developed using the R package lme4. Continuous variables are reported as median and interquartile range (IQR), and categorical variables are expressed as numbers and percentages (%). The log-rank test was used to compare the Kaplan–Meier (KM) curves.

## Results

### Patients

A total of 10,070 female TNBC patients with complete follow-up records who met the inclusion criteria were included in this study. The overall mortality rate was 19.0% (95% CI, 19.2–19.9%) over a median (IQR) follow-up time of 60 (42–48) months. The median (IQR) age was 58 (49–67) years, and the median (IQR) tumor size was 21 (13–32) mm. In total, 1,310 patients (15.2%) were in the non-chemotherapy group, and 7,310 (84.8%) received chemotherapy. The baseline clinical characteristics of all patients are presented in Table 1.

**Table 1:** Patients.

	No chemotherapy (*n* = 1,568)	Chemotherapy (*n* = 8,520)
Age, median (IQR), y	70 (61–78)	56 (47–64)
Tumor size, median (IQR), mm	11 (6–20)	23 (15–34)
Lymph node-positive, median (IQR), number	2 (1–4)	4 (2–10)
Race–white	1,196 (76.3)	6,016 (70.6)
Marriage–Married	748 (47.7)	4,991 (58.6)
Income–Higher than $70,000	562 (35.8)	2,853 (33.5)
Laterality–Right	766 (48.9)	4,089 (48.0)
Grade		
G1	82 (5.2)	63 (0.7)
G2	446 (28.4)	1,175 (13.8)
G3	1,034 (65.9)	7,249 (85.1)
G4	6 (0.4)	33 (0.4)
Histology		
Ductal	1,521 (97.0)	8,325 (97.7)
Lobular	26 (1.7)	73 (0.9)
Ductal-lobular	20 (1.3)	121 (1.4)
Location		
Upper outer quadrant	686 (43.8)	3,701 (43.4)
Upper inner quadrant	218 (13.9)	1,299 (15.2)
Lower outer quadrant	125 (8.0)	634 (7.4)
Lower inner quadrant	99 (6.3)	559 (6.6)
Central/overlapping	427 (27.2)	2,246 (26.4)
Nipple/axillary tail	13 (0.8)	81 (1.0)
T stage		
T1	1,202 (76.7)	3,679 (43.2)
T2	301 (19.2)	3,798 (44.6)
T3	46 (2.9)	719 (8.4)
T4	19 (1.1)	322 (3.7)
N stage		
N0	1,392 (88.8)	5,238 (61.5)
N1	110 (7.0)	2,219 (26.0)
N2	35 (2.2)	666 (7.8)
N3	31 (2.0)	395 (4.6)
TNM stage		
IA	1,147 (73.2)	2,865 (33.6)
IB	14 (0.9)	119 (1.4)
IIA	254 (16.2)	2,581 (30.3)
IIB	66 (4.2)	1,419 (16.7)
IIIA	43 (2.7)	887 (10.4)
IIIB	13 (0.8)	254 (3.0)
IIIC	31 (2.0)	395 (4.6)
Surgical type		
Breast-conserving surgery	632 (40.3)	3,757 (44.1)
Mastectomy	936 (59.7)	4,763 (55.9)

### Model performance

The testing set contained 2,573 patients, while the external testing sets included 1,468 patients diagnosed in 2010. All performance indicators were calculated in the testing and external testing sets with a preset time horizon of 10 years. The detailed model performance is demonstrated in Table 2.

**Table 2:** Model performance.

Model	IBS^a^	IBS^b^	HR	IPTW-adjusted HR	RD (%)	IPTW-adjusted RD (%)	DRMST (month)	IPTW-adjusted DRMST (month)
Performance in the testing set								
SNB	**0.09 (0.08–0.10)**	**0.10 (0.09–0.11)**	**0.62 (0.48–0.79)**	**0.68 (0.50–0.94)**	**7.61 (2.82–12.43)**	**9.06 (4.15–13.91)**	**6.48 (2.57–9.99)**	**7.08 (2.89–10.98)**
BITES	0.10 (0.09–0.12)	0.11 (0.10–0.13)	0.75 (0.60–9.99)	0.77 (0.60–9.99)	4.56 (0.20–8.92)	4.51 (0.25–8.77)	3.73 (0.18–7.29)	1.35 (−2.12–4.13)
CMHE	0.18 (0.15–0.21)	0.17 (0.15–0.18)	0.66 (0.52–0.84)	0.72 (0.82–0.98)	1.81 (−2.38–6.01)	6.38 (0.78–12.00)	1.38 (−2.04–4.79)	1.99 (−2.66–5.89)
DeepSurv	0.14 (0.11–0.17)	0.15 (0.12–0.17)	1.65 (1.29–2.12)	1.83 (1.06–3.14)	−6.98 (−11.2 t o −2.77)	−4.2 (−9.0 to −0.41)	−5.83 (−9.27 to −2.39)	−1.33 (−4.46–2.22)
CPH	0.11 (0.09–0.13)	0.11 (0.10–0.12)	0.80 (0.64–0.99)	0.71 (0.55–0.98)	3.79 (0.80–6.78)	4.55 (1.66–7.44)	3.08 (0.63–5.53)	2.22 (0.48–4.79)
NCCN			1.31 (0.99–1.72)	0.83 (0.50–1.38)	6.62 (1.82–11.40)	8.15 (3.38–12.90)	−5.56 (−8.12 to −2.93)	2.78 (−0.90–6.45)
Performance in the external testing set								
SNB	**0.12 (0.11–0.13)**	**0.12 (0.11–0.13)**	**0.54 (0.41–0.72)**	**0.53 (0.32–8.60)**	7.18 (1.85–12.30)	12.90 (6.99–19.01)	2.87 (1.29–3.92)	5.54 (1.36–8.61)
BITES	0.12 (0.10–0.14)	0.13 (0.12–0.14)	0.69 (0.53–0.89)	0.73 (0.55–0.89)	4.37 (−1.8–10.60)	4.08 (−1.7–9.8)	4.31 (−0.24–8.85)	2.64 (−1.40–6.21)
CMHE	0.18 (0.16–0.20)	0.16 (0.15–0.17)	0.61 (0.48–0.80)	0.62 (0.42–0.96)	6.04 (1.59–11.20)	13.00 (6.83–19.20)	1.73 (−2.49–5.95)	**8.15 (5.28–13.24)**
DeepSurv	0.16 (0.13–0.19)	0.16 (0.14–0.19)	1.44 (1.08–1.91)	1.51 (0.95–2.39)	−7.12 (−13.10 to −1.13)	−6.71 (−12.50 to −0.90)	−2.20 (−6.29–1.87)	−1.17 (−6.88–4.28)
CPH	0.13 (0.10–0.15)	0.12 (0.11–0.13)	0.74 (0.58–0.95)	0.68 (0.50–0.93)	5.23 (0.74–9.73)	5.40 (1.11–9.72)	**4.08 (0.94–7.23)**	5.39 (2.50–8.22)
NCCN			1.52 (1.12–2.08)	0.75 (0.46–1.22)	**8.16 (3.34–13.00)**	**8.11 (3.34–13.00)**	−3.71 (−7.04 to −0.37)	5.29 (1.39–9.88)

SNB, Self-Normalizing Balanced individual treatment effect for survival data; BITES, Balanced Individual Treatment Effect for Survival data; CMHE, Cox Mixtures with Heterogeneous Effects; CPH, Cox proportional hazards model; NCCN, National Comprehensive Cancer Network treatment guidelines. IBS, integrated Brier score; HR, hazard ratio; RD, 10-year risk difference; DRMST, the difference in the 10-year restricted mean survival time. ^a^Integrated Brier score in the non-chemotherapy group; ^b^Integrated Brier score in the chemotherapy group. The bold font indicates that the model performs best in this metric.

Patients with pT1–3pN0–1mi and tumor > 1 cm or with pN+ are recommended to receive adjuvant chemotherapy according to the NCCN guidelines.

The integrated Brier score (IBS) was calculated to measure the error between the model predicted and actual survival distributions within both factual and counterfactual situations (31), which can reflect the purity and success of counterfactual phenotyping (22). In both testing (IBS in the non-chemotherapy group (IBS^a^): 0.09, 95% CI, 0.08–0.10; IBS in the chemotherapy group (IBS^b^): 0.10, 95% CI, 0.09–0.11) and external testing sets (IBS^a^: 0.12, 95% CI, 0.11–0.13; IBS^b^: 0.12, 95% CI, 0.11–0.13), we observed that SNB had the best discrimination, followed by BITES (IBS^a^ in the testing set: 0.10, 95% CI, 0.09–0.12; IBS^b^ in the testing set: 0.11, 95% CI, 0.10–0.13; IBS^a^ in the external testing set: 0.12, 95% CI, 0.10–0.14; IBS^b^ in the external testing set: 0.13, 95% CI, 0.12–0.14).

The models predicted patients’ factual and counterfactual survival purely based on baseline covariates. Then, the ITEs and subsequent treatment recommendations were obtained. The metrics of interest lie in how much survival advantages can be gained by following model recommendations, which can be reflected by evaluating the protective effect of the Consis. group compared to the Inconsis. group. We set the metrics that decide the performance of the model to those corrected with IPTW, as they were largely unaffected by other prognostic factors. We also compared the NCCN guidelines with the models. The NCCN guidelines recommend TNBC patients with pT1–3pN0–1mi and tumor>1 cm or with pN+ to receive chemotherapy (8). Patients whose actual treatment was consistent with the NCCN guidelines were compared to those who were inconsistent.

In the testing set, the following SNB recommendation resulted from the most significant survival enhancement (IPTW-adjusted HR: 0.68, 95% CI, 0.50–0.94; IPTW-adjusted RD: 9.06, 95% CI, 4.15–13.91; IPTW-adjusted DRMST: 7.08, 95% CI, 2.89–10.98). CPH (IPTW-adjusted HR: 0.71, 95% CI, 0.55–0.98; IPTW-adjusted RD: 4.55, 95% CI, 1.66–7.44; IPTW-adjusted DRMST: 2.22, 95% CI, 0.48–4.79) and CMHE (IPTW-adjusted HR: 0.72, 95% CI, 0.82–0.98; IPTW-adjusted RD: 6.38, 95% CI, 0.78–12.00; IPTW-adjusted DRMST: 1.99, 95% CI, −2.66–5.89) both ranked second. Following NCCN guidelines only reduced 10-year mortality (RD: 6.62, 95% CI, 1.82–11.40; IPTW-adjusted RD: 8.15, 95% CI, 3.38–12.90).

In the external testing set, SNB had the best HR (0.54, 95% CI, 0.41–0.72) and IPTW-adjusted HR (0.53, 95% CI, 0.32–8.60), while CMHE demonstrated the best IPTW-adjusted DRMST (8.15, 95% CI, 5.28–13.24). CPH had the best DRMST (4.08, 95% CI, 0.94–7.23); however, this advantage disappeared after IPTW correction. NCCN had the best IPTW-adjusted RD (RD: 8.16, 95% CI, 3.34–13.00; IPTW-adjusted RD: 8.11, 95% CI, 3.34–13.00). However, NCCN did not demonstrate a protective effect in multivariate metrics, such as HR (1.52, 95% CI, 1.12–2.08) and IPTW-adjusted HR (0.75, 95% CI, 0.46–1.22). Thus, SNB was the best treatment recommendation tool in both testing and external testing sets, which outperformed other models and the NCCN guidelines.

**Figure 2:**
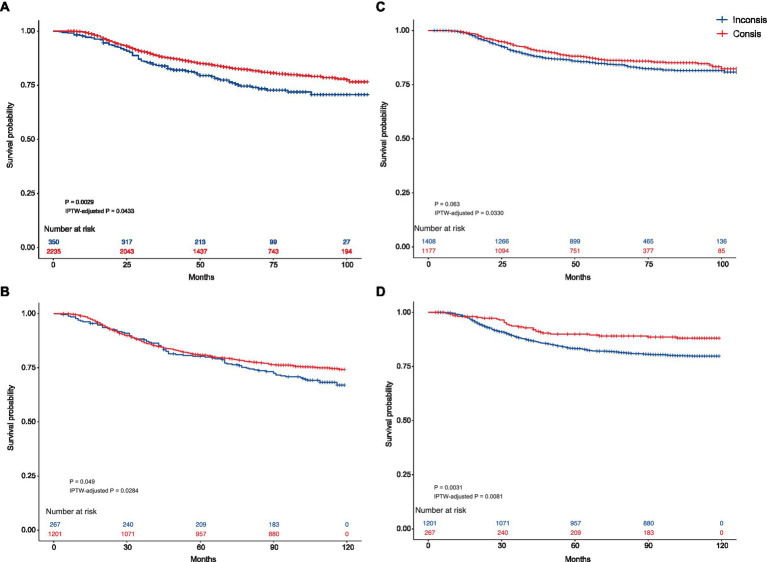
The Kaplan–Meier curves of the Consis. and Inconsis. groups. **(A)** Kaplan–Meier curves comparing the overall survival between the Consis. group and the Inconsis. group in the testing set. The *p*-values are derived from the log-rank test (*p* = 0.0029) and IPTW-adjusted log-rank test (IPTW-adjusted *p* = 0.0433). **(B)** Kaplan–Meier curves comparing the overall survival between the Consis. group and the Inconsis. group in the external testing set. The *p*-values are from the log-rank test (*p* = 0.0490) and the IPTW-adjusted log-rank test (IPTW-adjusted *p* = 0.0284). **(C)** Kaplan–Meier curves comparing the breast cancer-specific survival between the Consis. group and the Inconsis. group in the testing set. The *p*-values are derived from the log-rank test (*p* = 0.063) and the IPTW-adjusted log-rank test (IPTW-adjusted *p* = 0.0330). **(D)** Kaplan–Meier curves comparing the breast cancer-specific survival between the Consis. group and the Inconsis. group in the external testing set. The *p*-values are from the log-rank test (*p* = 0.0031) and the IPTW-adjusted log-rank test (IPTW-adjusted *p* = 0.0081).

The KM curves of the SNB-recommended Consis. group versus Inconsis. group in the testing and external testing sets are presented in Figures 2A,B, while that of breast cancer-specific survival (BCSS) is demonstrated in Figures 2C,D. Better OS of the Consis. group in the testing (*P* of log-rank test = 0.0029; *P* of IPTW-adjusted log-rank test = 0.0433) and external testing (*P* of log-rank test = 0.0490; *P* of IPTW-adjusted log-rank test = 0.0284) sets was visualized. The BCSS of the Consis. group was better than that of the Inconsis. group with degraded performance (Testing set: *P* of log-rank test = 0.0630; *P* of IPTW-adjusted log-rank test = 0.0330; External testing set: *P* of log-rank test = 0.0031; *P* of IPTW-adjusted log-rank test = 0.0081).

**Figure 3:**
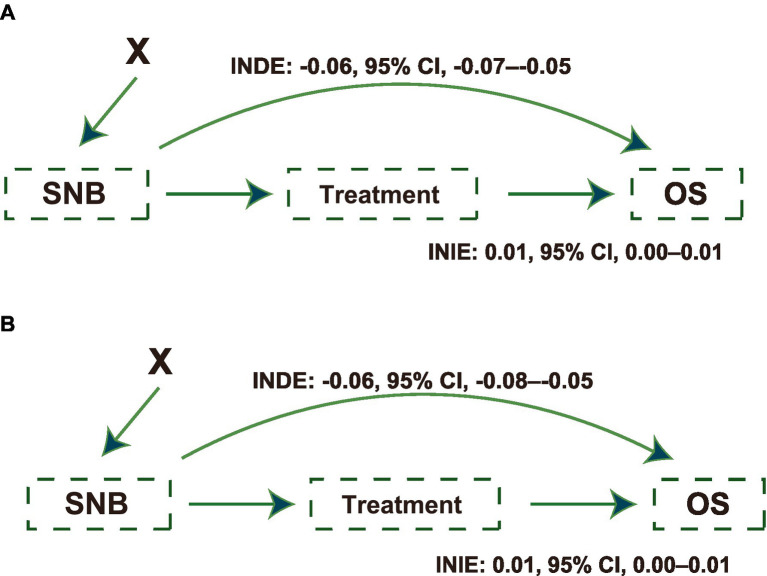
Causal paths illustrating the effects of Self-Normalizing Balanced (SNB) individual treatment effect for survival data on survival outcomes. **(A)** Causal path diagram showing the effects of SNB on overall survival (OS) in the testing set. The diagram quantifies the Interventional Natural Direct Effect (INDE: −0.06; 95% CI, −0.07 to −0.05) and Interventional Natural Indirect Effect (INIE: 0.01; 95% CI, 0.00 to 0.01), illustrating the direct and mediated impacts on OS, adjusted for baseline features as confounders. **(B)** The causal path diagram in the external testing set similarly details the SNB’s impact on OS. Displays the INDE (−0.06; 95% CI, −0.08 to −0.05) and INIE (0.01, 95% CI, 0.00 to 0.01), highlighting the robustness of SNB’s effect across different testing scenarios. X indicates patients’ baseline features, which were adjusted as intermediate confounders.

Whether the protective effect of SNB was affected by an imbalance in treatment proportions is also of interest. Thus, the interventional natural direct effect (INDE) was calculated to cut off the effect of treatment variables on OS improvement, which was proposed by Diaz et al. (32). We treated the treatments (chemotherapy and surgical type) as a mediator and adjusted for baseline features. The INDE and interventional natural indirect effect (INIE) are presented in Figures 3A,B for testing and external testing sets, respectively. These values were presented as the slope of a linear regression. SNB recommendation had a direct effect on OS improvement (INDE in the testing set: −0.06, 95% CI, −0.07 to −0.05; INIE in the testing set: 0.01, 95% CI, 0.00–0.01; INDE in the external testing set: −0.06, 95% CI, −0.08 to −0.05; INIE in the external testing set: 0.01, 95% CI, 0.00–0.01), which was not affected by treatments.

The standardized mean difference (SMD) before and after IPTW correction is shown in Supplementary Figure S1A (testing set) and Supplementary Figure S1B (external testing set). Covariates were balanced after IPTW with between-group SMDs smaller than 0.1 (33).

### Treatment heterogeneity

**Figure 4:**
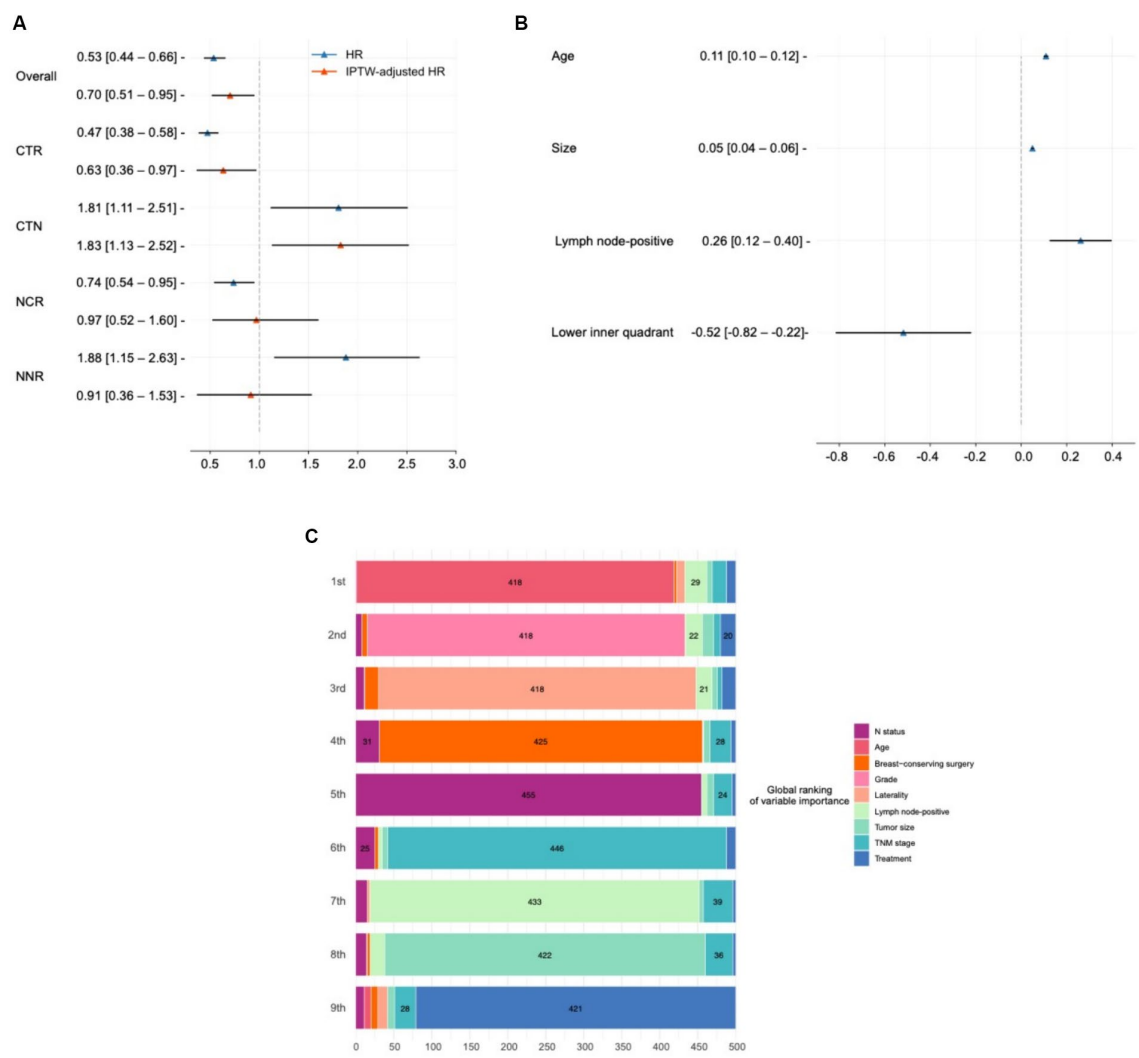
Model interpretation of treatment effects and variables impact. **(A)** Average treatment effect (ATE) and treatment heterogeneity present the hazard ratios (HRs) and IPTW-adjusted HRs demonstrating the ATE of chemotherapy across different patient groups, including chemotherapy recommended (CTR) and not recommended (CNR) groups classified by SNB, as well as groups classified according to the NCCN guidelines (NCR and NNR). **(B)** Interpretation of model recommendation behavior shows beta values from a mixed-effect linear regression predicting individual treatment effects from covariates with the region as a random effect in the combined testing and external testing sets. These beta values indicate the impact of one unit increase in covariates on the survival time difference over 10 years between chemotherapy and no chemotherapy. **(C)** Interpretation of overall output using SurvSHAP(t). Visualizes the aggregation of the eight most important variables influencing treatment decision, based on the Sharpley values derived from the SurvSHAP(t) analysis over 500 observations. This diagram details how variables rank in terms of importance across multiple observations.

Treatment heterogeneity can be captured by the presence of very different average treatment effects (ATEs) in different subgroups, indicating that patients with different characteristics respond heterogeneously to the same treatment. Patients were divided into chemotherapy recommended (CTR) and chemotherapy not recommended (CNR) groups based on whether chemotherapy was recommended by SNB. Similarly, patients were also divided into NCCN recommend chemotherapy (NCR) and NCCN not recommend chemotherapy (NNR) groups determined by whether the patients met the NCCN guidelines. This analysis was done using a combined population of testing and external testing sets. Figure 4A demonstrates the HR and IPTW-adjusted HR of chemotherapy in these subgroups.

In the overall population, chemotherapy had a protective effect (IPTW-adjusted HR: 0.70, 95% CI, 0.51–0.95). This effect was enhanced when SNB predicted a positive ITE of chemotherapy (IPTW-adjusted HR in the CTR group: 0.63, 95% CI, 0.36–0.97). Conversely, chemotherapy turned out to be a risk effect in the CTN group (IPTW-adjusted HR: 1.83, 95% CI, 1.13–2.52).

The treatment heterogeneity was also recognized by NCCN (HR in the NCR group: 0.74, 95% CI, 0.54–0.95; HR in the NNR group: 1.88, 95% CI, 1.15–2.63). However, the results turned negative after IPTW correction (IPTW-adjusted HR in the NCR group: 0.97, 95% CI, 0.52–1.60; IPTW-adjusted HR in the NNR group: 0.91, 95% CI, 0.36–1.53).

### Deep learning-based treatment insights

The ITE values reflect the difference in RST between chemotherapy and non-chemotherapy, indicating the additional survival time of an individual patient receiving chemotherapy. Considering that patients were from different regions, we derived a mixed-effect linear regression that predicts ITEs from the covariates with reporting region set as random effects, which was done in the combined population of testing and external testing sets. In such cases, the beta values obtained can be interpreted as follows: when other features hold, the presence of this covariate or an increase of one unit causes the difference in the survival time within 10 years of chemotherapy over no chemotherapy to increase beta. These results are presented in Figure 4B.

It was found that, for every 1 mm increase in the size of a patient’s tumor, chemotherapy increases their survival time by a relative 0.05 (95% CI, 0.04–0.06) months over 10 years. Similarly, patients with advanced age (0.11, 95% CI, 0.10–0.12) and more positive lymph nodes (0.26, 95% CI, 0.12–0.40) resulted in better efficacy of chemotherapy. Patients with tumors in the upper inner quadrant were not recommended for chemotherapy (−0.52, 95% CI, −0.82 to −0.22). Subsequently, we conducted a subgroup analysis (Supplementary Table S1), with the efficacy of chemotherapy increases with age and tumor size.

### Model interpretation

We used SurvSHAP(t) to interpret the functional output of SNB, the first method introduced to date that can provide a time-dependent interpretation with a solid theoretical basis (34). Figure 4C visualizes aggregating the eight most important variables, sorted by aggregated Sharpley values and rankings over 500 observations. The horizontal bars represent the number of observations where the importance of the variable is ranked first, second, and so on, indicated by the given color. The “treatment” variable indicated the effect of using different risk networks and baseline hazards.

Age was deemed the most important prognostic factor in 418 samples, followed by histologic grade, laterality, and surgical type.

One patient was randomly selected from the testing set and analyzed with SNB, shown in Supplementary Figure S2. With the help of SNB, the survival probability during different treatment plans was clearly demonstrated. Based on the predicted survival distribution, various indicators of survival advantages can then be calculated, including differences in mortality, time at risk, and RST, to facilitate the users’ self-directed choice of a more appropriate treatment plan.

## Discussion

Determining which TNBC patients require adjuvant chemotherapy involves multifactorial considerations (11) and remains controversial (7). Avoiding overtreatment and individualizing treatment plans for patients are key to achieving precision medicine.

Therefore, in this study, we carefully evaluated SNB, which outperformed state-of-the-art models, widely used alternatives, real-world physician choices, and NCCN guidelines. After diligently correcting for biases, following SNB recommendations can halve patients’ 10-year mortality rate, significantly outperforming alternative approaches. In addition to OS, following SNB guidance significantly improved BCSS in TNBC patients. We observed that the NCCN guidelines resulted in a positive RD and successfully identified treatment heterogeneity; however, these findings were statistically significant only in univariate metrics not corrected by IPTW. Treatment selection often needs to consider complex feature interactions rather than being based on fixed guidelines (25), and our study demonstrated that DL models are well suited to accomplish this, as clearly evidenced by the stronger protective effect of SNB than the NCCN guidelines.

Artificial intelligence-guided intervention studies provide the opportunity to gain insights from DL-based treatments by interpreting model recommendations associated with ITE values. We accounted for and excluded the influence of confounding factors on treatment recommendations by keeping other covariates constant. Thus, compared to the conclusions from traditional methods, these results are virtually independent of confounding factors and are quantifiable, which provides an essential basis for visualizing the impact of baseline characteristics on the relative efficacy of chemotherapy.

Consistent with previous studies, we found that for every 1 mm increase in the size of a patient’s tumor (10, 35), chemotherapy resulted in a relative extension of their 10-year survival time by 0.05 months. Other features, including number of positive lymph nodes (8) and age (11), also significantly affect chemotherapy efficacy. Interestingly, we found that chemotherapy was not recommended for patients with tumor sites located in the inner lower quadrant. The relationship between chemotherapy efficacy and tumor location has rarely been discussed, while past studies have only mentioned that TNBC patients with tumor sites located in the inner lower quadrant have a poorer prognosis after receiving neoadjuvant chemotherapy (36, 37). Therefore, this result can only be used as a reference at present. The reliability of it needs to be further investigated with more data and more teams, which may provide clinicians with new treatment ideas.

In response to the widely publicized effect of age and tumor size, the results of our subgroup analyses are also consistent with the findings of current mainstream, authoritative studies. Patients with TNBC over 65 years of age were more likely to benefit from adjuvant chemotherapy (38), which was not found to be statistically different in relatively younger patients. This indicates a greater need to incorporate multiple factors in the younger population when making final treatment decisions. In addition, consistent with the NCCN guidelines, adjuvant chemotherapy improves survival in TNBC with tumor size greater than 1 cm (8). However, for patients with smaller tumor sizes, it is important to combine other factors to make the final decision (39).

Developing a survival benefit visualization tool is essential for enabling patients and physicians to make informed treatment decisions. This tool facilitates the visual comparison of expected outcomes from various treatment options via a graphical treatment recommendation system, which incorporates multiple individual and comparative survival metrics. However, crafting personalized treatment plans and executing visual prognostic analyses remains challenging in practice (12, 40). Most current models utilize patient characteristics to generate prognostic factors, yet these are often influenced by biases from different treatments (41). The SNB model has the potential to overcome these challenges by more accurately demonstrating individual outcomes following various treatment regimens. With SNB, patients and physicians can visualize the anticipated effects of different treatment choices, playing a pivotal role in the final decision-making process. In addition, the cost of treatment is also a key consideration for patients, and by considering the cost of incorporating various therapies in the future, the SNB can help patients filter out the most cost-effective and optimal solution. It is also worth mentioning that for patients who have lost the ability to make decisions on their own, the SNB can greatly help their families to objectively analyze the pros and cons of different treatment options. All these predict the future application of the DL model in clinical treatment. In the future, improvements in data quality and including more disease types will refine these models further, laying a strong foundation for the entire field of precision medicine.

### Limitations

Due to database restrictions, we could not access some important features, such as Ki67, TILs, BRCA status, the presence of positive margins, and patient treatment switching or termination. Given that such biological factors are highly important prognostic markers concerning the survival of TNBC patients, we strongly advocate conducting further research to delve into this topic, contingent upon the availability of pertinent data regarding this information. Although the absence of crucial data above can affect treatment outcomes, the model’s usefulness is expected to increase as the variety and quality of variables improve. The generalizability of our results is limited by using a single database when training and testing the model. This approach may introduce biases associated with demographic and geographic diversity that do not accurately reflect the entire patient population. Despite our best efforts to control for bias in the data, reliance on retrospective data inherently limits the ability to control variables and interventions that were not initially recorded, which may introduce unmeasured bias and inconsistent observation times (42). Subsequent studies are recommended to test the protective effect of the model through randomized control trials, prospective cohort studies, or target trial emulation (43). Furthermore, considering the subjective nature of patient decisions, it is vital to include additional prognostic factors, such as complications and survival benefits, with anticipated improvements in database variables facilitating this comprehensive approach.

## Conclusion

To the best of our knowledge, this is the first study to develop individualized adjuvant chemotherapy recommendations for TNBC patients using DL. Moreover, our study confirms SNB’s potential to enhance clinical treatment decision-making and offer quantitative treatment insights. The model predicted enhanced chemotherapy efficacy in patients with older age, larger tumors, and a higher number of positive lymph nodes.

## Data availability statement

The datasets presented in this study can be found in online repositories. The names of the repository/repositories and accession number(s) can be found below: https://seer.cancer.gov/index.html.

## Ethics statement

The studies involving humans were approved by the national cancer institution. The studies were conducted in accordance with the local legislation and institutional requirements. Written informed consent for participation was not required for this study in accordance with national legislation and institutional requirements.

## Author contributions

XY: Conceptualization, Data curation, Formal analysis, Funding acquisition, Investigation, Methodology, Project administration, Resources, Software, Supervision, Validation, Visualization, Writing – original draft, Writing – review & editing. RV: Conceptualization, Formal analysis, Investigation, Methodology, Project administration, Resources, Software, Supervision, Validation, Visualization, Writing – original draft, Writing – review & editing. PG: Conceptualization, Data curation, Formal analysis, Investigation, Methodology, Resources, Software, Supervision, Validation, Visualization, Writing – original draft, Writing – review & editing. AS: Conceptualization, Methodology, Project administration, Writing – review & editing. MH: Formal analysis, Investigation, Methodology, Validation, Writing – review & editing. ZZ: Formal analysis, Investigation, Methodology, Writing – review & editing. YQ: Data curation, Investigation, Validation, Writing – review & editing. YC: Formal analysis, Investigation, Resources, Writing – review & editing. KS: Conceptualization, Formal analysis, Methodology, Writing – review & editing.

## References

[1] Zannetti A. Breast Cancer: from pathophysiology to novel therapeutic approaches 2.0. Int J Mol Sci. (2023) 24. doi: 10.3390/ijms24032542, PMID: 36768866 PMC9916418

[2] Bray F Ferlay J Soerjomataram I Siegel RL Torre LA Jemal A. Global cancer statistics 2018: GLOBOCAN estimates of incidence and mortality worldwide for 36 cancers in 185 countries. CA Cancer J Clin. (2018) 68:394–424. doi: 10.3322/caac.21492, PMID: 30207593

[3] Leon-Ferre RA Goetz MP. Advances in systemic therapies for triple negative breast cancer. BMJ. (2023) 381:e071674. doi: 10.1136/bmj-2022-071674 37253507

[4] Hammond ME Hayes DF Dowsett M Allred DC Hagerty KL Badve S. American Society of Clinical Oncology/College of American Pathologists guideline recommendations for immunohistochemical testing of estrogen and progesterone receptors in breast cancer (unabridged version). Arch Pathol Lab Med. (2010) 134:e48–72. doi: 10.5858/134.7.e48, PMID: 20586616

[5] Nedeljković M Damjanović A. Mechanisms of chemotherapy resistance in triple-negative breast cancer-how we can rise to the challenge. Cells. (2019) 8. doi: 10.3390/cells8090957, PMID: 31443516 PMC6770896

[6] Lin NU Vanderplas A Hughes ME Theriault RL Edge SB Wong YN. Clinicopathologic features, patterns of recurrence, and survival among women with triple-negative breast cancer in the national comprehensive cancer network. Cancer. (2012) 118:5463–72. doi: 10.1002/cncr.27581, PMID: 22544643 PMC3611659

[7] Anurag M Jaehnig EJ Krug K Lei JT Bergstrom EJ Kim BJ. Proteogenomic markers of chemotherapy resistance and response in triple-negative breast cancer. Cancer Discov. (2022) 12:2586–605. doi: 10.1158/2159-8290.CD-22-0200, PMID: 36001024 PMC9627136

[8] Gradishar WJ Moran MS Abraham J Abramson V Aft R Agnese D. NCCN guidelines® insights: breast cancer, version 4.2023. J Natl Compr Cancer Netw. (2023) 21:594–608. doi: 10.6004/jnccn.2023.0031, PMID: 37308117

[9] Carbajal-Ochoa W Bravo-Solarte DC Bernal AM Anampa JD. Benefit of adjuvant chemotherapy in lymph node-negative, T1b and T1c triple-negative breast cancer. Breast Cancer Res Treat. (2024) 203:257–69. doi: 10.1007/s10549-023-07132-6, PMID: 37833449

[10] An X Lei X Huang R Luo R Li H Xu F. Adjuvant chemotherapy for small, lymph node-negative, triple-negative breast cancer: a single-center study and a meta-analysis of the published literature. Cancer. (2020) 126:3837–46. doi: 10.1002/cncr.32878 32710666

[11] Li Y Ma R Chen H Pu S Xie P He J. A novel risk-scoring system to identify the potential population benefiting from adjuvant chemotherapy for node-negative TNBC patients with tumor size less than 1 cm. Front Oncol. (2022) 12:788883. doi: 10.3389/fonc.2022.788883, PMID: 35814418 PMC9260021

[12] Lei L Candès EJ. Conformal inference of counterfactuals and individual treatment effects. J R Stat Soc B (Stat Methodol). (2020) 83:911–38. doi: 10.1111/rssb.12445

[13] Yao L Chu Z Li S Li Y Gao J Zhang A. A survey on causal inference. ACM Trans Knowl Discov Data. (2020) 15:1–46. doi: 10.1145/3444944

[14] Zhu E Chen Z Ai P Wang J Zhu M Xu Z. Analyzing and predicting the risk of death in stroke patients using machine learning. Front Neurol. (2023) 14:1096153. doi: 10.3389/fneur.2023.1096153, PMID: 36816575 PMC9936182

[15] She Y Jin Z Wu J Deng J Zhang L Su H. Development and validation of a deep learning model for non-small cell lung Cancer survival. JAMA Netw Open. (2020) 3:e205842. doi: 10.1001/jamanetworkopen.2020.5842, PMID: 32492161 PMC7272121

[16] Zhu E Shi W Chen Z Wang J Ai P Wang X. Reasoning and causal inference regarding surgical options for patients with low-grade gliomas using machine learning: a SEER-based study. Cancer Med. (2023) 12:20878–91. doi: 10.1002/cam4.6666, PMID: 37929878 PMC10709720

[17] Hankey BF Ries LA Edwards BK. The surveillance, epidemiology, and end results program: a national resource. Cancer Epidemiol Biomarkers Prev. (1999) 8:1117–21. 10613347

[18] von Elm E Altman DG Egger M Pocock SJ Gøtzsche PC Vandenbroucke JP. The strengthening the reporting of observational studies in epidemiology (STROBE) statement: guidelines for reporting observational studies. Lancet. (2007) 370:1453–7. doi: 10.1016/S0140-6736(07)61602-X 18064739

[19] Künzel SR Sekhon JS Bickel PJ Yu B. Metalearners for estimating heterogeneous treatment effects using machine learning. Proc Natl Acad Sci USA. (2019) 116:4156–65. doi: 10.1073/pnas.1804597116, PMID: 30770453 PMC6410831

[20] Schrod S Schäfer A Solbrig S Lohmayer R Gronwald W Oefner PJ. BITES: balanced individual treatment effect for survival data. Bioinformatics. (2022) 38:i60–7. doi: 10.1093/bioinformatics/btac221, PMID: 35758796 PMC9235492

[21] Katzman JL Shaham U Cloninger A Bates J Jiang T Kluger Y. DeepSurv: personalized treatment recommender system using a cox proportional hazards deep neural network. BMC Med Res Methodol. (2018) 18:24. doi: 10.1186/s12874-018-0482-1, PMID: 29482517 PMC5828433

[22] Nagpal C Goswami M Dufendach KA Dubrawski AW: Counterfactual phenotyping with censored time-to-events. Proceedings of the 28th ACM SIGKDD Conference on Knowledge Discovery and Data Mining; (2022).

[23] Li F Morgan KL Zaslavsky AM. Balancing covariates via propensity score weighting. J Am Stat Assoc. (2014) 113:390–400. doi: 10.1080/01621459.2016.1260466

[24] Johansson FD Shalit U Kallus N Sontag DA. Generalization bounds and representation learning for estimation of potential outcomes and causal effects. J Mach Learn Res. (2020) 23:1–50.

[25] Pan H Wang J Shi W Xu Z Zhu E. Quantified treatment effect at the individual level is more indicative for personalized radical prostatectomy recommendation: implications for prostate cancer treatment using deep learning. J Cancer Res Clin Oncol. (2024) 150:67. doi: 10.1007/s00432-023-05602-4 38302801 PMC10834597

[26] Klambauer G Unterthiner T Mayr A Hochreiter S: Self-normalizing neural networks. In: Neural Information Processing Systems; (2017).

[27] Feydy J Séjourné T Vialard F-X Amari SI Trouvé A Peyré G. Interpolating between optimal transport and MMD using Sinkhorn divergences. In: International Conference on Artificial Intelligence and Statistics; (2018).

[28] Groenwold RHH Palmer TM Tilling K. To adjust or not to adjust? When a "confounder" is only measured after exposure. Epidemiology. (2021) 32:194–201. doi: 10.1097/EDE.0000000000001312, PMID: 33470711 PMC7850592

[29] Zhu E Zhang L Liu Y Ji T Dai J Tang R. Determining individual suitability for neoadjuvant systemic therapy in breast cancer patients through deep learning. Clin Transl Oncol. (2024). doi: 10.1007/s12094-024-03459-8, PMID: 38678522

[30] Forrest WF Alicke B Mayba O Osinska M Jakubczak M Piatkowski P. Generalized additive mixed modeling of longitudinal tumor growth reduces bias and improves decision making in translational oncology. Cancer Res. (2020) 80:5089–97. doi: 10.1158/0008-5472.CAN-20-0342, PMID: 32978171

[31] Kvamme H Borgan R: The brier score under administrative censoring: Problems and solutions; (2019).

[32] Díaz I Hejazi NS Rudolph KE Laan MJVD. Non-parametric efficient causal mediation with intermediate confounders. Biometrika. (2020) 108:627–41. doi: 10.1093/biomet/asaa085

[33] Austin PC. Some methods of propensity-score matching had superior performance to others: results of an empirical investigation and Monte Carlo simulations. Biom J. (2009) 51:171–84. doi: 10.1002/bimj.200810488, PMID: 19197955

[34] Krzyzi'nski M Spytek M Baniecki H Biecek P. SurvSHAP(t): time-dependent explanations of machine learning survival models. Knowl Based Syst. (2022) 262:110234. doi: 10.1016/j.knosys.2022.110234

[35] Ren YX Hao S Jin X Ye FG Gong Y Jiang YZ. Effects of adjuvant chemotherapy in T1N0M0 triple-negative breast cancer. Breast. (2019) 43:97–104. doi: 10.1016/j.breast.2018.11.011, PMID: 30529406

[36] Song X Zhang Q. The poor prognosis of lower-inner quadrant breast cancer in patients who received neoadjuvant chemotherapy. Ann Palliat Med. (2020) 9:1859–71. doi: 10.21037/apm-20-1140, PMID: 32576015

[37] Chang JH Jeon W Kim K Shin KH Han W Noh DY. Prognostic significance of inner quadrant involvement in breast cancer treated with neoadjuvant chemotherapy. J Breast Cancer. (2016) 19:394–401. doi: 10.4048/jbc.2016.19.4.394, PMID: 28053627 PMC5204045

[38] Liu X Yue S Huang H Duan M Zhao B Liu J. Risk stratification model for predicting the overall survival of elderly triple-negative breast cancer patients: a population-based study. Front Med (Lausanne). (2021) 8:705515. doi: 10.3389/fmed.2021.705515, PMID: 34621757 PMC8490672

[39] Gupta RK Roy AM Gupta A Takabe K Dhakal A Opyrchal M. Systemic therapy De-escalation in early-stage triple-negative breast cancer: dawn of a new era? Cancers (Basel). (2022) 14. doi: 10.3390/cancers14081856, PMID: 35454764 PMC9025008

[40] Zhu E Zhang L Wang J Hu C Pan H Shi W. Deep learning-guided adjuvant chemotherapy selection for elderly patients with breast cancer. Breast Cancer Res Treat. (2024) 205:97–107. doi: 10.1007/s10549-023-07237-y, PMID: 38294615

[41] Di Ieva A. AI-augmented multidisciplinary teams: hype or hope? Lancet. (2019) 394:1801. doi: 10.1016/S0140-6736(19)32626-1 31699402

[42] VanderWeele TJ. Principles of confounder selection. Eur J Epidemiol. (2019) 34:211–9. doi: 10.1007/s10654-019-00494-6, PMID: 30840181 PMC6447501

[43] Hernán MA Wang W Leaf DE. Target trial emulation: a framework for causal inference from observational data. JAMA. (2022) 328:2446–7. doi: 10.1001/jama.2022.21383 36508210

