# Supplementary material for: Personalized chemotherapy selection for patients with triple-negative breast cancer using deep learning
Source: Front Med (Lausanne). 2024 Jun 20;11:1418800. doi: 10.3389/fmed.2024.1418800 (PMC11222643; doi:10.3389/fmed.2024.1418800)
Supplement: Supplementary file 1 [file Data_Sheet_1.docx]

Table S1: Subgroup analyses regarding age and tumor size

|  | Hazard ratio (95% CI) | IPTW-adjusted hazard ratio (95% CI) |
| --- | --- | --- |
| Age ≤ 50, years | 1.88 (1.41–2.92) | 1.25 (0.65–1.96) |
| 50 < Age ≤ 65, years | 1.41 (0.94–2.11) | 0.83 (0.46–1.51) |
| Age > 65, years | 0.81 (0.64–1.01) | 0.58 (0.45–0.74) |
| Tumor size ≤ 5, mm | 1.46 (1.13–2.64) | 1.19 (0.94–1.36) |
| 5 < Tumor size ≤ 100, mm | 0.74 (0.62–0.89) | 0.88 (0.49–1.22) |
| Tumor size > 100, mm | 0.33 (0.17–0.54) | 0.43 (0.12–0.78) |

The hazard ratio was calculated to measure the protective effect of chemotherapy within these subgroups. CI, confidence interval; IPTW, inverse probability treatment weighting adjusts for other demographic and tumor characteristics.

Figure S1: The standardized mean difference before and after inverse probability treatment weighting.

A: The standardized mean difference before and after inverse probability treatment weighting (IPTW) in the testing set. B: The standardized mean difference before and after IPTW in the external testing set. The standardized mean differences are all below the threshold of 0.1, indicating adequate balance between groups post-IPTW.

Figure S2: The individual survival distribution predicted by model.

Figure S2 illustrates the predicted survival probabilities and restricted survival times (RST) for a randomly selected patient from the testing set under different treatment plans: Adjuvant Chemotherapy (AdCT) and Non-Adjuvant Chemotherapy (Non-AdCT). Survival probabilities at 5 years are shown for both treatment scenarios. Additionally, the figure displays the time at risk (death rate at 50%) and restricted survival times over a 5-year period.
